# CNS cell-derived exosome signatures as blood-based biomarkers of neurodegenerative diseases

**DOI:** 10.3389/fnins.2024.1426700

**Published:** 2024-06-20

**Authors:** Calvin Park, Jonathan S. Weerakkody, Raphael Schneider, Sheng Miao, David Pitt

**Affiliations:** ^1^Columbia University Irving Medical Center, Columbia University, New York, NY, United States; ^2^Yale School of Medicine, Yale University, New Haven, CT, United States; ^3^Division of Neurology, St. Michael’s Hospital, Toronto, ON, Canada

**Keywords:** exosomes, extracellular vesicles, central nervous system, neurodegeneration, neuroinflammation, blood-based biomarkers

## Abstract

Molecular biomarkers require the reproducible capture of disease-associated changes and are ideally sensitive, specific and accessible with minimal invasiveness to patients. Exosomes are a subtype of extracellular vesicles that have gained attention as potential biomarkers. They are released by all cell types and carry molecular cargo that reflects the functional state of the cells of origin. These characteristics make them an attractive means of measuring disease-related processes within the central nervous system (CNS), as they cross the blood–brain barrier (BBB) and can be captured in peripheral blood. In this review, we discuss recent progress made toward identifying blood-based protein and RNA biomarkers of several neurodegenerative diseases from circulating, CNS cell-derived exosomes. Given the lack of standardized methodology for exosome isolation and characterization, we discuss the challenges of capturing and quantifying the molecular content of exosome populations from blood for translation to clinical use.

## Introduction

**FIGURE 1:**
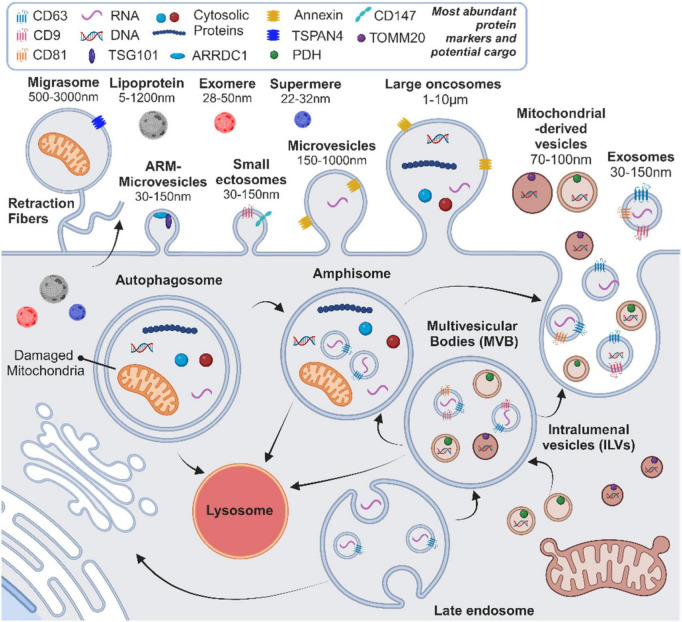
Current overview of the heterogeneity and biogenesis pathways for extracellular vesicles (EVs) and non-vesicular nanoparticles (NVEPs), highlighting key protein markers, potential cargo components, and size ranges. ARM, arrestin domain-containing protein 1-mediated. Created with BioRender.com.

Extracellular vesicles (EVs) are a diverse group of membranous structures, classified according to their biogenesis into several types: exosomes, derived from the endosomal compartment; microvesicles and ectosomes, formed by outward budding of the plasma membrane; apoptotic bodies, released from dying cells; oncosomes, originating from cancerous cells; and recently discovered migrasomes, vesicles produced during cell migration (Colombo et al., 2014; Thompson et al., 2016; Jeppesen et al., 2023; Zhang X. et al., 2023). Additionally, variants such as arrestin domain-containing protein 1-mediated (ARM) microvesicles and mitochondrial-derived EVs, as well as emerging non-vesicular extracellular nanoparticles (NVEPs), such as lipoproteins, exomeres and supermeres, further extend the diversity of particles released by cells (Neuspiel et al., 2008; Nabhan et al., 2012; Jeppesen et al., 2023; Figure 1). Since the assignment of EVs to specific subtypes requires definitive demonstration of their biogenesis pathways, with corroborative minimal experimental requirements, the International Society of Extracellular Vesicles (ISEV) recommends use of “extracellular vesicle” as the generic term for particles released by cells that are enclosed by a lipid bilayer (Welsh et al., 2024). For further specificity, operational terms to characterize EV types, including descriptions of size and physical characteristics, biochemical composition and cell of origin, can be used (Welsh et al., 2024). In this review, we use the terms “exosomes” or “EVs” interchangeably as appropriate to discussions of vesicle functions or as reported by the studies discussed.

Exosomes form by inward budding of the endosomal membrane to create multivesicular bodies (MVBs) containing intraluminal vesicles (ILVs). This formation is regulated by endosomal sorting complex required for transport (ESCRT) proteins, including CD9, CD63, and CD81, as well as ESCRT-independent mechanisms (Colombo et al., 2014; Thompson et al., 2016). MVBs fuse with the cell membrane, or first with autophagosomes to form amphisomes, through a tightly regulated process, which is followed by the release of ILVs as exosomes into the extracellular space and circulation (Colombo et al., 2014; Hornung et al., 2020; Ganesan and Cai, 2021; Figure 1). Exosomes carry a diverse collection of lipids, proteins and nucleic acids, which are present in their lipid bilayer or lumen, and are released in greater numbers following cellular activation and/or stress (Colombo et al., 2014; Thompson et al., 2016). Moreover, the molecular content of exosomes reflects the biological state of the cell of origin and may, therefore, provide insight into the phenotypic changes occurring in tissues affected by disease processes (Colombo et al., 2014; De Toro et al., 2015; Thompson et al., 2016). Exosomes are taken up by other cells and deposit their molecular content through endocytic mechanisms or direct fusion with the plasma membrane (Colombo et al., 2014). This deposition of bioactive material into recipient cells makes exosomes key intercellular messengers that regulate processes such as immune responses, wound healing, tissue regeneration and disease processes such as tumor progression (Colombo et al., 2014; De Toro et al., 2015; Thompson et al., 2016; Hettich et al., 2020; Hornung et al., 2020; Wan et al., 2022). While exosomes have the potential to interact with any cell type, there is no consensus regarding the natural targets of specific exosome populations or the main mechanisms of uptake by recipient cells and subsequent intracellular content delivery (Mathieu et al., 2019). It has recently been demonstrated that, at physiologic doses, EV-mediated surface protein signaling has a more potent effect on recipient cells than EV cargo, suggesting that cell surface signaling may represent the primary mechanism of EV-mediated effects on target tissues (Hagey et al., 2023). However, given the established effects of deposited EV cargo on intracellular processes (Zhao et al., 2016; Hermann et al., 2024), further work is needed to clarify the influence of different EV-cell interactions on recipient cell responses.

The regulatory role of exosomes and other EVs in the central nervous system (CNS) consists of maintaining myelination, glial cell function, neuronal trophic support and synaptic plasticity by mediating communication among cells (Budnik et al., 2016; Thompson et al., 2016). In neurodegenerative disorders, including Alzheimer’s disease (AD), Parkinson’s disease (PD), multiple system atrophy (MSA), amyotrophic lateral sclerosis (ALS), frontotemporal dementia (FTD), and Creutzfeldt-Jakob disease (CJD), the precise functions of exosomes have been under investigation. They have been shown to play both neurodegenerative and neuroprotective roles in these pathologies. In AD, exosomes from neurons and glial cells disseminate pathogenic amyloid-β and tau proteins, promoting their aggregation and disruption of vital neuronal processes including axonal transport (Rajendran et al., 2006; Dinkins et al., 2014; Joshi et al., 2014, 2015; Liu et al., 2019). Exosomes have also been suggested to counter neuronal loss through transmission of neuroprotective factors and parallel uptake of their pathogenic protein content by microglia (Yuyama et al., 2012, 2014; Malm et al., 2016). In PD and MSA, exosomes contribute to the dissemination and aggregation of misfolded α-synuclein and have been implicated in defective endosomal-lysosomal protein transport underlying neuronal loss (Lee et al., 2005; Emmanouilidou et al., 2010; Danzer et al., 2012; Grey et al., 2015; Ngolab et al., 2017; Teixeira et al., 2021; Mavroeidi et al., 2022). As in AD, exosomes may also confer neuroprotection in PD by transferring misfolded proteins and toxins from neurons to glial cells for degradation and supporting neuronal metabolic activity (Lee et al., 2008; Tomlinson et al., 2015). In FTD and ALS, TDP-43 has been shown to be deposited into cells and removed via exosomes (Nonaka et al., 2013; Feiler et al., 2015; Iguchi et al., 2016). SOD1, another pathogenic protein found in patients with ALS, is also misfolded and disseminated among cells via exosomes (Gomes et al., 2007; Grad et al., 2014). In CJD, exosomes are known to carry pathogenic PrP^Sc^ protein, implicating them in the accumulation and propagation of misfolded prions (Fevrier et al., 2004; Vilette et al., 2015; Guo et al., 2016; Hartmann et al., 2017; López-Pérez et al., 2021). Thus, exosomes have pathologic and protective functions in neurogenerative conditions, which continues to be an active area of investigation. The roles of exosomes in demyelinating diseases such as multiple sclerosis (MS) and neuromyelitis optica spectrum disorder (NMOSD), however, remain poorly characterized. These diseases involve both peripheral immune mechanisms leading to inflammatory demyelination and, in the case of MS, to secondary neurodegenerative processes (Nylander and Hafler, 2012; Bermel, 2017; Ponath et al., 2018; Wingerchuk and Lucchinetti, 2022). Some studies suggest that CNS and immune cell-derived exosomes promote immune cell infiltration and demyelination (Jy et al., 2004; Verderio et al., 2012; Xie et al., 2023), but these potential roles require additional substantiation.

Central nervous system-derived exosomes cross the blood–brain barrier (BBB) and enter the blood, providing an easily accessible window into cellular processes of the brain and spinal cord (Budnik et al., 2016; Chen et al., 2016; García-Romero et al., 2017). They can be isolated from blood based on size properties and surface markers indicating the cell type of origin (Chen et al., 2022). Analysis of these cell type-specific exosomes can identify disease-driven molecular changes that are otherwise obscured by measurements from exosomes released by other organ systems. Thus, CNS-originating exosomes and their content may present highly sensitive and specific, blood-based biomarkers of neurodegenerative diseases, for which precise means of quantifying underlying pathological processes are lacking (Gómez-Río et al., 2016). Research on EVs as markers of CNS pathologies has been influenced by investigations of EVs in cancers, which present a similar need to capture disease-specific cellular processes for accurate diagnostics and treatment monitoring (LeBleu and Kalluri, 2020). Explorations of blood-based, EV-derived markers for neoplasms have already identified highly specific and sensitive proteomic and transcriptomic signatures that reliably detect and classify multiple cancer types (Melo et al., 2015; Hoshino et al., 2020; Wang et al., 2022). Concurrently, putative EV biomarkers of neurodegenerative diseases have already been isolated from blood and cerebrospinal fluid (CSF) by many groups. Thus, quantification of CNS-originating exosomes and their molecular cargo presents a promising opportunity to discover new biomarkers of neurologic conditions and treatment response to improve on clinical assessments of disease. It should be noted that in addition to their potential as biomarkers, exosomes and other EVs are a promising therapeutic modality for neurodegenerative conditions (Hermann et al., 2024). The multimodal actions of specific cell type or lineage-derived EVs on recipient tissue, including modulation of gene expression, immune responses, cell metabolism and neuron-glia interactions, makes EVs potent mediators of neurologic recovery. Their therapeutic effects have already been demonstrated in mouse and non-human primate neurologic disease models, where they enhanced neuronal plasticity, angiogenesis and mitochondrial stability following disease onset or injury (Doeppner et al., 2015; Medalla et al., 2020; Peruzzotti-Jametti et al., 2021). Given the diverse signaling mechanisms involved in EV-based therapies, successful application of EVs to human subjects necessitates the determination of optimal cells of origin and isolation protocols to deliver specific molecular content (e.g., surface and luminal proteins, RNAs) tailored to the pathology of interest (Medalla et al., 2020; Wang et al., 2020). Moreover, the optimal route of administration (e.g., intravenous versus intracerebroventricular) of isolated EVs must be determined based on their known biodistribution, mechanisms of action (e.g., effects on peripheral versus CNS cells) and procedural risks, given that EVs may not cross the BBB in large enough quantities to exert direct effects on the CNS (Hermann et al., 2024). This process would benefit from minimally invasive measurements of treatment response, including molecular signatures from blood CNS cell type-derived exosomes.

In the following sections, we provide an update on recent progress made toward identifying protein and RNA biomarkers of neurodegenerative diseases and MS from CNS-derived exosomes.

## CNS cell-derived exosomes as biomarkers of neurodegenerative diseases

Current diagnostics and monitoring of neurodegenerative diseases are limited to clinical assessments, costly imaging techniques, such as magnetic resonance imaging (MRI) and positron emission tomography (PET) scans, and invasive lumbar punctures for CSF analysis (Gómez-Río et al., 2016; Hansson, 2021; Younas et al., 2022). As such, blood-based biomarkers are needed to improve the ease, cost and precision of these assessments. Novel peripheral biomarkers have utility beyond diagnostics and can be harvested as predictive, prognostic and disease progression measures at different clinical stages of neurodegenerative disorders. In addition, they may be of utility in clinical trials to pre-select patients based on marker activity and to monitor therapeutic efficacy. Development of blood-based markers has met the challenge of variable findings and poor correlation with disease burden measured by current CSF or imaging markers. This likely reflects the compartmentalization of key pathogenic processes to the CNS via the BBB (Zetterberg and Schott, 2019; Ashton et al., 2020). Therefore, the ability of exosomes released by CNS cells to cross the BBB presents an opportunity to discover markers of CNS-specific disease processes directly from patient blood. Moreover, work is needed to determine whether CNS-derived exosomes in the blood represent a different population from those in the CNS parenchyma and CSF, which will help establish the specificity of putative exosomal markers. As with other biomarkers for neurodegenerative diseases, the use of exosomes to diagnose these conditions may pose ethical challenges, given their potential to identify these conditions in both pre-clinical and clinical stages in the absence of effective or accessible therapies. In the following subsections, we discuss progress toward the development of diagnostic neuron and glial cell-derived exosomal markers of neurodegenerative conditions.

### Neuron-derived exosomes

Neuron-derived EVs and their protein content have successfully been isolated from the blood of patients with neurodegenerative conditions by multiple groups. Notably, Goetzl et al. (2015a,b) pioneered the analysis of neuron-derived exosomes from the plasma and serum of subjects with AD, PD, FTD, and healthy controls (Shi et al., 2014, 2016; Fiandaca et al., 2015). They established techniques for the isolation of neuronal exosomes though immunoprecipitation with antibodies against axonal marker L1CAM and cell adhesion protein NCAM. Of note, L1CAM, NCAM and most CNS cell type markers are expressed by multiple other cell types in the periphery (Uhlen et al., 2010), suggesting that blood EVs isolated by these groups are enriched for but not exclusively CNS cell-derived EVs. These studies and subsequent investigations of neuron-derived EVs in various neurodegenerative conditions have been reviewed more extensively elsewhere (Hornung et al., 2020; Liu et al., 2022; Dutta et al., 2023; Wang et al., 2023; Xu et al., 2024).

Importantly, these studies revealed specific protein signatures in blood-derived, L1CAM-positive EVs for AD, PD, atypical Parkinsonian syndromes and FTD. In AD patients, significant elevations of amyloid-β-peptide (Aβ)42 and phosphorylated tau as well as differential levels of lysosomal and synaptic proteins were reported in blood-derived L1CAM-positive exosomes compared to healthy controls, with high predictive power for development of AD and correlation with CSF levels (Fiandaca et al., 2015; Goetzl et al., 2015a,b, 2016a; Winston et al., 2016; Jia et al., 2019). In PD patients, α-synuclein and tau from blood-derived L1CAM-positive exosomes were elevated compared to healthy controls, correlating with CSF levels and disease severity (Shi et al., 2014, 2016; Zhao et al., 2019). Moreover, α-synuclein from neuronal exosomes was higher in MSA compared to PD and could reliably distinguish these diseases when used in combination with α-synuclein levels in oligodendroglial exosomes (Dutta et al., 2021; Taha et al., 2023). Recently, Meloni et al. (2023) showed that L1CAM-positive neuronal EVs contained elevated levels of oligomeric α-synuclein in PD compared to atypical Parkinsonian syndromes while Tau aggregates showed the opposite trend, demonstrating good classification power for PD, progressive supranuclear palsy (PSP) and corticobasal degeneration (CBD). Of note, most of these studies were case-control or cross-sectional in design, often with fewer than 50 patients per study group and without separate validation cohorts or follow-up investigations utilizing larger sample sizes. Thus, the validity of these proposed markers and their temporal relationship with different stages of disease remain to be determined.

There has been debate concerning the presence of L1CAM on the surface of EVs, given multiple processed forms of the protein and recent evidence showing that L1CAM is not associated with EV fractions from plasma and CSF (Norman et al., 2021). Other groups that attempted to isolate L1CAM-positive EVs from plasma demonstrated that EVs indeed expressed L1CAM (Dutta et al., 2021, 2023; Gomes and Witwer, 2022; Aguilar et al., 2023). Thus, there is conflicting evidence regarding the presence of L1CAM on EVs and whether or not it can reliably be used as a marker of neuron-derived EVs (Gomes and Witwer, 2022). Most studies to date have not utilized other markers to isolate neuron-derived EVs in parallel to L1CAM-positive EVs for direct comparison of their molecular cargos. Such efforts, in addition to quantification of putative markers in L1CAM-positive EVs from CSF and brain tissue, would clarify the neuronal specificity L1CAM and if multiple distinct populations of neuron-derived EVs exist. Several groups have recently employed high-throughput methods of analyzing the protein content of neuron-derived EVs from patient blood using Tandem Mass Tag-based quantitative proteomics or liquid chromatography-tandem mass spectrometry. Zhong et al. (2021) showed that complement component 7 (C7) and Zyxin (ZYX) were significantly increased and decreased, respectively, over the course of progression from mild cognitive impairment (MCI) to AD, as corroborated using a larger, independent cohort. Upon further validation, these two markers could present a promising means to evaluate cognitive decline prior to AD onset. Arioz et al. (2021) identified an increase in hemoglobin proteins in L1CAM-positive exosomes of 20 AD patients compared to 23 healthy controls. Anastasi et al. (2021) identified 23 proteins related to PD, including those of the ubiquitin-proteasome system, DJ-1/PARK7, Clusterin, Amyloid P component, Gelsolin, and CXCL12, which have previously been reported as candidate circulating biomarkers of PD (Zhao et al., 2019; Jiang et al., 2020). However, this study analyzed only four PD patients and four healthy controls as proof-of-concept for a novel immunoplate-based method of isolating L1CAM-positive EVs following serial centrifugation, thus requiring further validation (Anastasi et al., 2021).

The RNA content of neuron-derived EVs has also been under investigation. Of note, while there is a wealth of studies measuring RNA in total EVs from the blood of patients with neurodegenerative diseases, as discussed elsewhere (Liu et al., 2019, 2022; Sproviero et al., 2022; Bagyinszky et al., 2023; Dutta et al., 2023; Xu et al., 2024), there are fewer studies for neuronal and other CNS-derived EVs (Cha et al., 2019; Banack et al., 2020, 2022; Serpente et al., 2020; Zou et al., 2020; Durur et al., 2022; Li et al., 2022a,b; Pounders et al., 2022; Aguilar et al., 2023; Dunlop et al., 2023). This likely reflects poor reproducibility of RNA findings from total plasma, serum and CSF EVs due to unstandardized EV isolation techniques, use of different downstream RNA measurement techniques and variations in the patient populations studied (Liu et al., 2022; Dutta et al., 2023). Still, some newer studies have reported RNA signatures from neuron-derived EVs in AD, PD, FTD, and ALS.

Durur et al. (2022) utilized RNA sequencing to analyze L1CAM-positive EVs in AD compared to healthy control patients, reporting 10 differentially expressed miRNAs between the two groups and high diagnostic utility of let-7e-5p. Pounders et al. (2022) also applied RNA sequencing to L1CAM-positive plasma EVs, showing that miRNA-122 and miRNA-3591 were downregulated in AD compared to healthy controls and FTD, while miRNA-181c was downregulated in FTD compared to healthy controls. Moreover, they demonstrated high congruence between plasma and CSF-derived L1CAM-positive EVs using paired samples, supporting that the cargo of neuron-derived EVs in blood reflects CNS-intrinsic processes (Pounders et al., 2022). Serpente et al. (2020) analyzed miRNA from L1CAM-positive plasma neuronal EVs from AD patients and healthy controls using high-throughput polymerase chain reaction (PCR), showing upregulation of miR-23a-3p, miR-223-3p and miR-190-5p, and downregulation of miR-100-3p in AD compared to controls. Moreover, miR-23a-3p, miR-223-3p and miR-100-3p were not significantly dysregulated in the total plasma EV population, while miR-190a-5p was only detected in neuronal EVs, supporting the ability of cell type-specific EVs to uncover molecular changes intrinsic to the CNS (Serpente et al., 2020). Zou et al. (2020) demonstrated that Linc-POU3F3 was upregulated in L1CAM-positive plasma neuronal exosomes of PD patients compared to healthy controls, positively correlating with PD severity. Aguilar et al. (2023) reported a panel of 29 differentially expressed small RNAs in PD compared to matched healthy controls, several of which have previously been implicated as circulating biomarkers of PD or detected in PD brain tissue. These studies used various methods for isolating neuron-derived EVs and quantifying their RNA content, with substantial variability in sample sizes and findings, necessitating validation with a standardized approach.

From ALS patients, Banack et al. (2020, 2022) isolated plasma L1CAM-positive neuronal EVs and demonstrated a highly reproducible signature of eight dysregulated miRNAs compared to healthy controls through repeated studies, with up to 50 ALS and 50 healthy control samples. They showed significant differences in the expression of these eight miRNAs among total, L1CAM-positive and L1CAM-negative EV populations, supporting that enrichment for neuron-derived EVs contributes to the reproducibility of their miRNA signature (Dunlop et al., 2023). Of note, these miRNA findings do not align with results from an earlier high-throughput microarray analysis of plasma L1CAM-positive EVs in ALS, possibly due to the smaller sample size and EV isolation by gradient centrifugation versus polymer-based precipitation used in the earlier study (Katsu et al., 2019).

### Glial cell-derived exosomes

Compared to neuron-derived EVs, there have been fewer investigations of blood-based, glial-derived EVs for markers of neurodegenerative conditions, as summarized extensively elsewhere (Hornung et al., 2020; Dutta et al., 2023; Wang et al., 2023). Initial studies of astrocyte-derived EVs from plasma by Goetzl et al. (2016b,2018) analyzed the protein content of GLAST-positive exosomes. They notably demonstrated that BACE-1, soluble amyloid precursor protein (sAPP)β, multiple complement proteins and the inflammatory cytokines IL-6, TNF-α, and IL-1β were significantly higher in astrocyte-derived exosomes from patients with AD than controls, while Aβ42, GDNF, septin-8, and complement regulatory proteins were downregulated (Goetzl et al., 2016b,^2018^). Higher levels of proteins, including BACE-1, γ-secretase and sAPPβ, were found in astrocyte versus neuron-derived exosomes in AD, FTD and control samples (Goetzl et al., 2016b). Importantly, these findings have demonstrated molecular profiles enriched in astrocytic exosomes in AD but have not been supported by validation using larger patient cohorts, as with the discussed findings in neuron-derived EVs.

Several studies have also investigated blood-based, glial-derived EVs in PD and related disorders. Dutta et al. (2021) demonstrated that α-synuclein levels in myelin oligodendrocyte glycoprotein (MOG)-positive oligodendrocyte and L1CAM-positive neuron-derived exosomes from blood could together distinguish MSA from PD with high sensitivity and specificity (Taha et al., 2023). Yu et al. (2020) had previously isolated CNPase-positive oligodendrocyte-derived EVs from plasma, demonstrating significantly lower concentrations of these EVs in MSA compared to PD and healthy controls. Interestingly, they did not observe different levels of α-synuclein among these groups, unlike Dutta et al. (2021, 2023), which may be attributable to the different oligodendrocyte EV populations isolated (Yu et al., 2020). Nevertheless, oligodendrocyte-derived EVs are of particular interest in MSA, where α-synuclein predominantly accumulates in oligodendrocytes (Beyer and Ariza, 2007). Ohmichi et al. (2019) quantified the plasma levels of GLAST-positive, oligodendrocyte-myelin glycoprotein (OMG)-positive and SNAP25-positive astrocyte, oligodendrocyte and neuron-derived exosomes, respectively, from PD, MSA, PSP, and healthy control patients. They reported that oligodendrocyte and astrocyte-derived exosome levels were higher in early PD stages compared to healthy controls and increased at a more advanced stage. Moreover, the ratio of astrocyte and oligodendrocyte-derived exosomes to neuron-derived exosomes correlated significantly with PD severity, while the levels of oligodendrocyte-derived exosomes and their ratio with neuron-derived exosomes correlated with motor disability in MSA with predominant parkinsonism (Ohmichi et al., 2019). Together, these studies support that the cargo and quantity of astrocyte and oligodendrocyte-derived EVs from blood are candidate markers for PD and related disorders.

Recently, Kumar et al. (2023) showed significant dysregulation of multiple miRNAs in plasma-derived, TMEM119-positive microglial EVs among patients with AD, MCI conversion to AD (MCI-AD) and MCI compared with those of normal cognition. Notably, miR-29a-5p was decreased in AD, MCI-AD, and MCI compared with the normal controls, while miR-106b-5p and miR-132-5p were increased among AD and MCI-AD patients. Moreover, miR-132-5p and miR-125b-5p, which have previously been shown to regulate microglial activation as well as cytokine and chemokine release, were able to predict AD (Kumar et al., 2023). To our knowledge, there are no other recent studies on RNA markers from blood-derived, glial cell EVs for AD, PD, and other neurodegenerative disorders. As summarized above, exploration of RNA in CNS-derived EVs has focused on neuron-derived EVs, likely due to the primary role that neuronal dysfunction and death play in these pathologies. However, glial cells, including astrocytes, have an increasingly appreciated role in these disorders (Bhandarkar et al., 2023), meriting future investigation of the RNA content of glial cell EVs.

## CNS cell-derived exosomes as biomarkers in multiple sclerosis

Work on CNS-originating peripheral blood EVs has extended to finding markers of MS to assess disease progression and response to treatment. Currently, secondary progressive MS (SPMS) is monitored through clinical assessments and MRI. The increasing number of drug trials and use of approved medications for SPMS face a need for easily accessible, peripheral biomarkers to monitor response to treatment (Mallik et al., 2014; Kappos et al., 2018; Behrangi et al., 2019). Progressive MS is thought to be driven by chronic glial cell activation and concomitant neurodegeneration (Rissanen et al., 2018; Kaunzner et al., 2019; Gillen et al., 2021). Thus, glial cell-derived blood exosomes present an opportunity to develop an objective measure of MS disease activity, while neuron-derived exosomal markers may primarily reflect the degree of subsequent neuronal damage (Kaunzner et al., 2019; Gillen et al., 2021; Thebault et al., 2022). Changes in glial cell activity and the degree of neuronal loss captured by these exosomes can be used to both monitor responses to therapies and identify predictive markers of treatment response for individualized disease management. Studies to date on protein and RNA biomarkers of MS from blood and CSF-derived EVs have focused predominantly on total EVs, as reviewed extensively elsewhere (D’Anca et al., 2021; Selmaj et al., 2022). While the small number of patients examined limit these studies, the predictive power of reported markers from total exosomes suggests that pathologic changes in CNS resident cells are reflected in their cargo.

Bhargava et al. (2021) have isolated GLAST-positive and L1CAM-positive astrocytic and neuronal EVs, respectively, from the plasma of patients with RRMS and progressive disease as well as healthy controls. The authors reported significantly lower levels of synaptopodin and synaptophysin in neuronal EVs from both RRMS and progressive MS patients compared to controls, as well as increased levels of multiple complement cascade components in astrocytic EVs from MS patients compared to controls. Moreover, this significant difference in complement components was not present in total EVs, and there was a strong inverse correlation between neuronal EV synaptic proteins and multiple astrocyte EV complement components (Bhargava et al., 2021). These findings support that analysis of CNS-derived exosome populations can reveal cell type-specific processes and associated biomarkers that are not apparent in total exosomes. From RRMS patients, Mazzucco et al. (2022) isolated plasma EVs originating from CNS endothelial cells based on co-expression of myelin and lymphocyte protein (MAL) as well as the pan-endothelial markers CD31, CD105, or CD144, with exclusion of lymphocyte or platelet-derived EVs based on CD3 and CD41 expression. They showed a significant increase in the plasma concentration of CNS endothelial-derived EVs in RRMS patients with active disease not treated with disease modifying therapy (DMT), compared to stable RRMS patients and healthy controls. These findings demonstrate that levels of circulating EVs can serve as markers of disease in addition to their content. In cognitively impaired compared to cognitively preserved patients with RRMS and progressive disease, Scaroni et al. (2022) recently showed elevated miR-150-5p and reduced let-7b-5p levels in plasma-derived, Isolectin B4-positive microglial/macrophage EVs. However, these differences were not associated with progressive disease, and several study drawbacks, including small sample size and variable patient disease characteristics, limit the interpretation of these results (Scaroni et al., 2022). However, the identification of a miRNA signature related to both synapse function and cognitive deficits merits future characterization of microglial EVs for markers of progressive MS.

## CNS cell-derived exosome isolation techniques and challenges

Multiple methods to isolate exosomes from patient plasma or serum have been utilized. The ISEV has published guidelines on reporting EV isolation and characterization approaches toward maximizing reproducibility of published results, given the heterogeneous methodologies utilized in EV studies to date (Welsh et al., 2024). More recently, to improve the reproducibility of blood EV research, the ISEV Blood EV Task Force extended these standards to blood, plasma and serum-derived EVs (Lucien et al., 2023). These methods include serial ultracentrifugation at increasing speeds, sucrose gradient centrifugation, polymer precipitation, immunoprecipitation and size-exclusion chromatography (Chen et al., 2022; Welsh et al., 2024).

In serial ultracentrifugation, plasma or serum samples are centrifuged at increasing speeds to sequentially remove cells, debris and larger vesicles until the desired EV populations are precipitated. Sucrose gradient centrifugation utilizes media of similar or lower density to the EVs of interest to isolate them by density or mass/size. In polymer-based precipitation, EVs are incubated with hydrophilic polymers that capture particles of interest by lowering their solubility, allowing for their subsequent collection via centrifugation. Immunoprecipitation utilizes antibodies immobilized onto solid matrices that bind to specific surface antigens on EVs of interest, which are then precipitated by centrifugation and eluted. In size-exclusion chromatography, EVs are passed through a stationary phase with resin that contains pores of a specific size, isolating particles of a desired diameter range based on retention time (Yang et al., 2020; Chen et al., 2022). It should be noted that none of these techniques can completely separate exosomes from other EVs. While several of these methods have been widely used over recent years, their ability to capture a high enough yield of CNS-derived EVs for molecular profiling remains a challenge, as neuron or glial cell-derived EVs alone comprise a small fraction of total circulating EVs (Hornung et al., 2020; Younas et al., 2022). Moreover, blood samples available often yield low volumes of plasma for EV purification.

Concerns over low yield from small starting volumes even apply to commonly used density-gradient ultracentrifugation, considered to be the gold standard of exosome isolation due to its high processing capacity, and to size-exclusion chromatography, which is often chosen due to its efficiency and relatively pure yield (Chen et al., 2022; Younas et al., 2022). Drawbacks of these methods include the large time requirement for centrifugation, contributing to decreased EV yield and integrity with repeated shear force, as well as high cost and inability to differentiate EV types when using chromatography. Moreover, such techniques lead to the co-isolation of NVEPs, which further compromises purity (Zhang et al., 2018; Jeppesen et al., 2019; Zhang Q. et al., 2023). Immunoprecipitation with antibodies against traditional ESCRT markers (e.g., CD63, CD9, and CD81) (Colombo et al., 2014; Thompson et al., 2016) presumably captures exosomes, but these markers have not been standardized and specific EV types and NVEPs can only be confirmed by definitive demonstration of their biogenesis pathways (Welsh et al., 2024). Moreover, total yield of EVs immunolabeled with ESCRT markers from serum or plasma is low, possibly attributable to the presence of these markers in different post-translationally modified forms and variable expression in exosomes and across samples (Hoshino et al., 2020; Mazzucco et al., 2022; Younas et al., 2022). Polymer-based methods of exosome precipitation have improved yield at the expense of purity and protein coating of exosomes and can be used prior to immunoprecipitation of cell type-specific exosomes (Goetzl et al., 2015a; Helwa et al., 2017; Dutta et al., 2021). While this approach has been widely employed, its performance in capturing desired EVs as compared to other means of isolating total EVs has not been tested.

Significant progress beyond standard ultracentrifugation and polymer-based isolation has been made in developing novel total exosome isolation methods. These methods include microfluidics, clustering-and-scattering (i.e., forming aggregates of EVs from plasma using a cationic polymer, followed by elution and purification of EVs from filtered aggregates), lipid-affinity based purification methods and antibody-conjugated nanowires, which have emerged as potential means to increase exosome yield from small volumes (Contreras-Naranjo et al., 2017; Lim et al., 2019; Kim et al., 2020; Younas et al., 2022; Weerakkody, 2024). qEV isolation (Izon Science) has emerged as an efficient, size-exclusion chromatography technique that yields high exosome quantities and purity compared to other commercially available methods, as validated by several studies using plasma and other biofluids (Stranska et al., 2018; Navajas et al., 2019; Almiñana et al., 2021; Veerman et al., 2021). In parallel, an automated exosome detection method via the ultrafast-isolation system (EXODUS) has been developed as a novel ultrafiltration technique to isolate exosomes from biofluids, with reported improvement in speed, cost, purity and yield compared to other exosome isolation methods (Chen et al., 2021). These new techniques require further assessment of their exosome yield and ease of implementation into clinical use.

Multiple methods can be employed to assess exosome yield and validate particle identity as well as cell type specificity. For exosome purity, commonly used methods include Western blots using antibodies against traditional ESCRT markers, as well as Nanoparticle Tracking Analysis (NTA) or Microfluidic Resistive Pulse Sensing (MRPS) to measure particle size and number. In addition to confirming the appropriate diameter and yield of isolated EVs, direct visualization of particle morphology and marker expression can be performed with transmission electron microscopy (TEM) and fluorescent immunolabeling under super-resolution microscopy (Lai et al., 2022). Cell type specificity of exosome populations can be demonstrated through enrichment of cell type markers (e.g., gamma-enolase for neurons, GFAP or AQP4 for astrocytes, and PLP for oligodendrocytes) using enzyme-linked immunosorbent assay (ELISA) and Western or dot blots (Hornung et al., 2020). Moreover, the molecular signatures of these peripherally isolated exosome populations can be compared to the same populations isolated from CSF and CNS tissue to further validate cell type specificity.

As discussed above, markers that distinguish different cell types within the CNS are typically expressed by multiple peripheral cell types. For instance, L1CAM is expressed not only by neurons, but also by melanocytes and cells of the urinary tract, whereas GLAST is expressed by astrocytes, platelets, epithelial cells and macrophages, among others (Uhlen et al., 2010). Thus, EVs that are identified through neuronal or glial cell markers in blood are enriched but not specific for these cell types. In contrast, oligodendrocyte-specific exosomes have been isolated by multiple groups using MOG, OMG, or CNPase antibodies (Ohmichi et al., 2019; Yu et al., 2020; Dutta et al., 2021). These markers are essentially exclusive to oligodendrocytes, although they are present at different stages of oligodendrocyte differentiation and may have varying availability on the surface of EVs (Trapp et al., 1988; Kuhn et al., 2019).

Isolating microglia-derived exosomes remains a challenge, as their defining homeostatic markers such as TMEM119 are downregulated in pathologies or expressed by other cell types (Prinz et al., 2017; Masuda et al., 2019; Jurga et al., 2020). Therefore, microglial exosomes are not easily distinguishable from those derived from peripheral monocytes, macrophages and other organ systems. Still, recent studies have demonstrated RNA and protein signatures in microglia-derived or enriched EVs isolated from patients with neurologic diseases. As discussed in the previous section, Kumar et al. (2023) characterized the miRNA content of TMEM119-positive EVs from the plasma of patients with AD and MCI. Several groups have isolated broader populations of microglial/macrophage EVs from blood or CSF, quantifying EV levels or their molecular content as relevant to the pathology of interest. Scaroni et al. (2022) identified a miRNA signature in Isolectin B4-positive microglial/macrophage EVs from the plasma of MS patients, identifying individuals with cognitive deficits, as summarized in the previous section. Guo et al. (2020) isolated CD11b-positive microglia/macrophage-derived exosomes from the CSF of PD and MSA patients as well as healthy controls, showing differential elevation of total and oligomeric α-synuclein among these groups, while Isolectin B4-positive microglial/macrophage EVs have been isolated from the CSF of MS patients to correlate EV levels with disease activity (Dalla Costa et al., 2021; Gelibter et al., 2021). Increased CNS specificity can be achieved with sequential immunoprecipitation using several cell type markers to eliminate non-CNS-derived exosomes expressing only one of the markers. However, such refinement may come at the cost of decreased exosome yield for downstream analysis. Another consideration is that expression of these markers can fluctuate with disease burden, as discussed above. Further work on how to reliably capture CNS-specific signatures is needed, possibly through novel cell type markers. The ability to capture CNS-originating exosome populations with precision may be enhanced by new single-vesicle and micro/nanofluidic isolation techniques, including droplet-based extracellular vesicle analysis (DEVA), which uses fluorescent microbead-based ELISA to capture single EVs of interest (Yang et al., 2022), track-etched magnetic nanopore (TENPO) sorting, which employs a chip with magnetic nanopores to capture immunomagnetically labeled EVs (Ko et al., 2018, 2020), and multiplexed antibody-based immunosequencing (Ko et al., 2021). These techniques have been reviewed more extensively elsewhere (Dutta et al., 2023).

Thus, current challenges in developing blood-based, CNS-derived exosome biomarkers include low yield of specific populations, uncertain CNS specificity of exosomes using standard neuronal and glial cell markers, and lack of protocol standardization for exosome purification and characterization. Comparisons among different approaches to isolating total exosomes from small volumes and subsequent purification of cell type specific populations will identify a method that produces the highest yield of CNS-derived exosomes while maintaining the highest specificity to the cells of interest. Given the precision, time and expertise required to minimize errors in this process, technical improvements are needed as a step toward maximizing reproducibility, minimizing variation between experimental batches and ultimately standardizing the optimal methodology.

Quantification of molecular content from exosome populations constituting a small fraction of total circulating exosomes and containing variable amounts of biologic material may pose a challenge even to sensitive techniques such as quantitative PCR (qPCR), RNA sequencing, proteomics approaches and ELISA. The detection threshold of available nucleic acid and protein expression assays, therefore, may be a limiting factor to development of these biomarkers, with potential inability to reproduce findings and non-specific detection of other circulating molecules (Chitoiu et al., 2020). RNA quantification technology continues to improve, with advances including methods targeted toward creation of reproducible RNA sequencing libraries from picogram amounts of total RNA from samples (SMARTer Stranded Total RNA-seq Kit – Pico Input Mammalian, Takara Bio) and nanotechnology techniques (Song et al., 2018, 2023). Moreover, sequencing results can be validated using digital PCR (dPCR) and droplet digital PCR (ddPCR), which offer more precision and reproducibility of measurements compared to standard qPCR for low starting levels of RNA (Vogelstein and Kinzler, 1999; Taylor et al., 2017). Ultrasensitive protein assay techniques such as electrochemiluminescence ELISA (ECLIA) and single-molecule array (Simoa) may also offer a more reliable means to quantify small amounts of proteins isolated from CNS-originating EVs (Song et al., 2013; Stefura et al., 2019).

**FIGURE 2:**
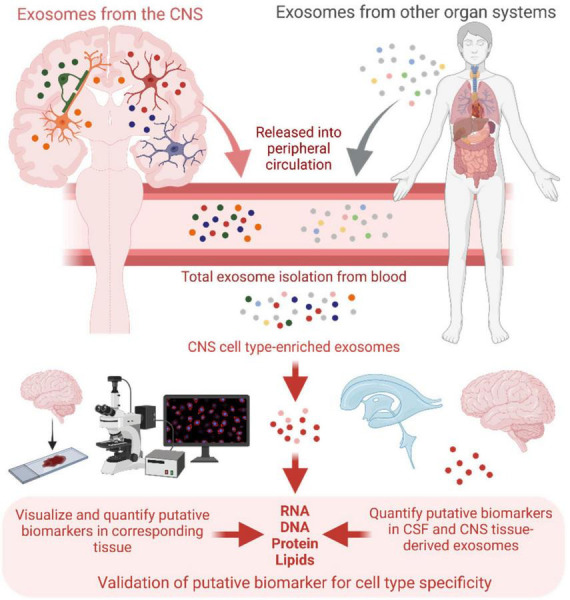
Schematic of central nervous system (CNS) cell type-enriched exosome isolation from patient blood, and validation of putative biomarker cell type specificity with exosomes isolated from corresponding cerebrospinal fluid (CSF) and CNS tissue samples, as well as through visualization of the putative biomarkers in relevant cell subtypes directly in tissue. Created with BioRender.com.

If increased purity of cell type-derived exosomes comes at the expense of molecular yield and accurate quantification, then less specific populations will need to be analyzed. One solution is to compare the molecular signatures of broader blood exosome populations enriched for the cell type-derived exosomes of interest, using less CNS-specific markers, with those of the same populations isolated from paired CSF samples. Because of its relation to the CNS, the CSF likely contains exosomes that are predominantly of brain and spinal cord origin, providing the most CNS-specific population of exosomes outside of those isolated directly from CNS tissue. Thus, putative nucleic acid or protein markers from broader peripheral exosome populations that show the same pattern of dysregulation in CSF-derived exosomes can be said to reflect CNS-intrinsic changes, which can be validated using corresponding CNS tissue-derived exosomes if available. Moreover, the CNS origin and pathologic significance of putative markers can be demonstrated by visualization in specific activated neuronal or glial subtypes in tissue using RNA *in situ* hybridization and immunofluorescence techniques (Figure 2). While this may overcome the need to isolate highly specific exosome populations, the availability of CSF and tissue samples could present a challenge. Development of newer technologies such as high-throughput single-vesicle transcriptomics and proteomics may overcome most or all of these limitations.

## Discussion

The ubiquitous release of exosomes by all cell types and reflection of parent cell phenotypes in their vesicular cargo suggests considerable potential for exosomes as a means of quantifying disease-specific processes. Moreover, the role of exosomes in mediating intercellular communication and spreading pathogenic material makes them ideal candidates for monitoring processes such as protein aggregation that underlie neurodegenerative pathophysiology.

In recent years, successful efforts to isolate and analyze CNS-derived EV populations from peripheral blood have further increased the appeal of exosomes as blood-based markers of CNS diseases. Multiple groups have demonstrated specific protein and RNA signatures from CNS-derived exosomes in AD, PD, and other conditions, thereby demonstrating the feasibility of this approach. At the same time, findings frequently vary among the discussed studies, especially for exosomal RNA content, and presumably due in part to the heterogeneity of methods for exosome isolation and molecular quantification, as well as variability in patient cohorts. Moreover, most results to date have not been replicated in large patient cohorts. As a result, numerous candidate markers have been reported, many of them intuitively plausible; however, their validity and reproducibility remain questionable.

While technical advances have allowed for characterization of CNS-specific exosomes from blood, commonly used purification strategies are costly, time-consuming and require expertise. Moreover, reliable quantification of exosome cargo can be limited by insufficient yield from small blood volumes, considering that CNS cell type-derived exosomes comprise only a small fraction of total circulating exosomes. Thus, strategies to optimize and standardize total and cell type-specific exosome retrieval as well as downstream molecular profiling techniques are needed. Novel exosome isolation techniques should be compared with commonly used methods such as ultracentrifugation and polymer-based precipitation in regard to blood-based, CNS cell type-derived exosome yield, reproducibility of downstream results using both high-throughput omics and targeted molecular assays such as qPCR and ELISA, and efficiency to select a protocol that can be applied universally to this field of research. Once a rigorously evaluated method of exosome isolation and characterization is established, current findings of candidate protein and RNA biomarkers from CNS-originating exosomes can be conclusively validated. Standardized validation steps for CNS cell type-derived exosome molecular signatures must be established and should ideally include evaluation of marker cell type specificity using the same exosome populations isolated from corresponding CSF and/or CNS tissue. Moreover, highly sensitive and precise assays for molecular quantification (e.g., ddPCR and ECLIA) should be employed to ensure reliable and consistent results. With reproducible findings, the performance of putative markers compared to standard clinical, laboratory and imaging tools for diagnosing and monitoring neurodegenerative diseases can be assessed in large-scale studies before approval for clinical use. Moreover, standardized methodology will allow markers of treatment response, including for EV-based therapies, to be developed and used by future clinical trials for novel neurodegenerative disease treatments. Thus, with appropriate optimization of protocols and validation of findings, blood-derived exosomes are poised to become precise markers of disease and potentially a practical means of quantifying neurologic processes associated with disease, which will enhance patient care and facilitate discovery of new therapies.

## Author contributions

CP: Conceptualization, Visualization, Writing – original draft, Writing – review & editing. JW: Visualization, Writing – review & editing. RS: Writing – review & editing. SM: Writing – review & editing. DP: Conceptualization, Funding acquisition, Project administration, Supervision, Visualization, Writing – original draft, Writing – review & editing.

## References

[B1] Aguilar M. Ebanks S. Markus H. Lewis M. Midya V. Vrana K. (2023). Neuronally enriched microvesicle RNAs are differentially expressed in the serums of Parkinson’s patients. *Front. Neurosci.* 17:1145923. 10.3389/fnins.2023.1145923 37483339 PMC10357515

[B2] Almiñana C. Rudolf Vegas A. Tekin M. Hassan M. Uzbekov R. Fröhlich T. (2021). Isolation and characterization of equine uterine extracellular vesicles: A comparative methodological study. *Int. J. Mol. Sci.* 22:979. 10.3390/ijms22020979 33478136 PMC7835857

[B3] Anastasi F. Masciandaro S. Carratore R. Dell’Anno M. Signore G. Falleni A. (2021). Proteomics profiling of neuron-derived small extracellular vesicles from human plasma: Enabling single-subject analysis. *Int. J. Mol. Sci.* 22:2951. 10.3390/ijms22062951 33799461 PMC7999506

[B4] Arioz B. Tufekci K. Olcum M. Durur D. Akarlar B. Ozlu N. (2021). Proteome profiling of neuron-derived exosomes in Alzheimer’s disease reveals hemoglobin as a potential biomarker. *Neurosci. Lett.* 755:135914. 10.1016/j.neulet.2021.135914 33901610

[B5] Ashton N. Hye A. Rajkumar A. Leuzy A. Snowden S. Suárez-Calvet M. (2020). An update on blood-based biomarkers for non-Alzheimer neurodegenerative disorders. *Nat. Rev. Neurol.* 16 265–284. 10.1038/s41582-020-0348-0 32322100

[B6] Bagyinszky E. Hulme J. An S. S. A. (2023). Studies of genetic and proteomic risk factors of amyotrophic lateral sclerosis inspire biomarker development and gene therapy. *Cells* 12:1948. 10.3390/cells12151948 PMC10417729 37566027

[B7] Banack S. A. Dunlop R. A. Cox P. A. (2020). An miRNA fingerprint using neural-enriched extracellular vesicles from blood plasma: Towards a biomarker for amyotrophic lateral sclerosis/motor neuron disease. *Open Biol.* 10:200116. 10.1098/rsob.200116 PMC7333885 32574550

[B8] Banack S. Dunlop R. Stommel E. Mehta P. Cox P. (2022). miRNA extracted from extracellular vesicles is a robust biomarker of amyotrophic lateral sclerosis. *J. Neurol. Sci.* 442:120396. 10.1016/j.jns.2022.120396 36081303

[B9] Behrangi N. Fischbach F. Kipp M. (2019). Mechanism of siponimod: Anti-inflammatory and neuroprotective mode of action. *Cells* 8:24. 10.3390/cells8010024 PMC6356776 30621015

[B10] Bermel R. (2017). Unravelling neurodegeneration in multiple sclerosis. *Lancet Neurol.* 16 764–766. 10.1016/S1474-4422(17)30302-2 28920874

[B11] Beyer K. Ariza A. (2007). Protein aggregation mechanisms in synucleinopathies: Commonalities and differences. *J. Neuropathol. Exp. Neurol.* 66 965–974. 17984679 10.1097/nen.0b013e3181587d64

[B12] Bhandarkar S. Qavi D. Park C. Pitt D. (2023). *Astrocytes in neuroinflammatory and neurodegenerative diseases.* Amsterdam: Elsevier.

[B13] Bhargava P. Nogueras-Ortiz C. Kim S. Delgado-Peraza F. Calabresi P. Kapogiannis D. (2021). Synaptic and complement markers in extracellular vesicles in multiple sclerosis. *Mult. Scler.* 27 509–518. 10.1177/1352458520924590 32669030 PMC7744427

[B14] Budnik V. Ruiz-Cañada C. Wendler F. (2016). Extracellular vesicles round off communication in the nervous system. *Nat. Rev. Neurosci.* 17 160–172. 10.1038/nrn.2015.29 26891626 PMC4989863

[B15] Cha D. Mengel D. Mustapic M. Liu W. Selkoe D. Kapogiannis D. (2019). miR-212 and miR-132 are downregulated in neurally derived plasma exosomes of Alzheimer’s patients. *Front. Neurosci.* 13:1208. 10.3389/fnins.2019.01208 31849573 PMC6902042

[B16] Chen C. Liu L. Ma F. Wong C. Guo X. Chacko J. (2016). Elucidation of exosome migration across the blood-brain barrier model in vitro. *Cell Mol. Bioeng.* 9 509–529. 10.1007/s12195-016-0458-3 28392840 PMC5382965

[B17] Chen J. Li P. Zhang T. Xu Z. Huang X. Wang R. (2022). Review on strategies and technologies for exosome isolation and purification. *Front. Bioeng. Biotechnol.* 9:811971. 10.3389/fbioe.2021.811971 35071216 PMC8766409

[B18] Chen Y. Zhu Q. Cheng L. Wang Y. Li M. Yang Q. (2021). Exosome detection via the ultrafast-isolation system: EXODUS. *Nat. Methods* 18 212–218. 10.1038/s41592-020-01034-x 33432243

[B19] Chitoiu L. Dobranici A. Gherghiceanu M. Dinescu S. Costache M. (2020). Multi-omics data integration in extracellular vesicle biology-utopia or future reality? *Int. J. Mol. Sci.* 21:8550. 10.3390/ijms21228550 33202771 PMC7697477

[B20] Colombo M. Raposo G. Théry C. (2014). Biogenesis, secretion, and intercellular interactions of exosomes and other extracellular vesicles. *Annu. Rev. Cell Dev. Biol.* 30 255–289. 25288114 10.1146/annurev-cellbio-101512-122326

[B21] Contreras-Naranjo J. C. Wu H.-J. Ugaz V. M. (2017). Microfluidics for exosome isolation and analysis: Enabling liquid biopsy for personalized medicine. *Lab Chip* 17 3558–3577. 28832692 10.1039/c7lc00592j PMC5656537

[B22] Dalla Costa G. Croese T. Pisa M. Finardi A. Fabbella L. Martinelli V. (2021). CSF extracellular vesicles and risk of disease activity after a first demyelinating event. *Mult. Scler.* 27 1606–1610. 10.1177/1352458520987542 33464186

[B23] D’Anca M. Fenoglio C. Buccellato F. Visconte C. Galimberti D. Scarpini E. (2021). Extracellular vesicles in multiple sclerosis: Role in the pathogenesis and potential usefulness as biomarkers and therapeutic tools. *Cells* 10:1733. 10.3390/cells10071733 34359903 PMC8303489

[B24] Danzer K. Kranich L. Ruf W. Cagsal-Getkin O. Winslow A. Zhu L. (2012). Exosomal cell-to-cell transmission of alpha synuclein oligomers. *Mol. Neurodegener.* 7:42. 10.1186/1750-1326-7-42 22920859 PMC3483256

[B25] De Toro J. Herschlik L. Waldner C. Mongini C. (2015). Emerging roles of exosomes in normal and pathological conditions: New insights for diagnosis and therapeutic applications. *Front. Immunol.* 6:203. 10.3389/fimmu.2015.00203 25999947 PMC4418172

[B26] Dinkins M. B. Dasgupta S. Wang G. Zhu G. Bieberich E. (2014). Exosome reduction in vivo is associated with lower amyloid plaque load in the 5XFAD mouse model of Alzheimer’s disease. *Neurobiol. Aging* 35 1792–1800. 24650793 10.1016/j.neurobiolaging.2014.02.012 PMC4035236

[B27] Doeppner T. Herz J. Görgens A. Schlechter J. Ludwig A. Radtke S. (2015). extracellular vesicles improve post-stroke neuroregeneration and prevent postischemic immunosuppression. *Stem Cells Transl. Med.* 4 1131–1143. 10.5966/sctm.2015-0078 26339036 PMC4572905

[B28] Dunlop R. A. Banack S. A. Cox P. A. (2023). L1CAM immunocapture generates a unique extracellular vesicle population with a reproducible miRNA fingerprint. *RNA Biol.* 20 140–148. 37042019 10.1080/15476286.2023.2198805 PMC10101655

[B29] Durur D. Tastan B. Ugur Tufekci K. Olcum M. Uzuner H. Karakülah G. (2022). Alteration of miRNAs in small neuron-derived extracellular vesicles of Alzheimer’s disease patients and the effect of extracellular vesicles on microglial immune responses. *J. Mol. Neurosci.* 72 1182–1194. 10.1007/s12031-022-02012-y 35488079

[B30] Dutta S. Hornung S. Kruayatidee A. Maina K. Del Rosario I. Paul K. (2021). α-Synuclein in blood exosomes immunoprecipitated using neuronal and oligodendroglial markers distinguishes Parkinson’s disease from multiple system atrophy. *Acta Neuropathol.* 142 495–511. 10.1007/s00401-021-02324-0 33991233 PMC8357708

[B31] Dutta S. Hornung S. Taha H. Bitan G. (2023). Biomarkers for Parkinsonian disorders in CNS-originating EVs: Promise and challenges. *Acta Neuropathol.* 145 515–540. 10.1007/s00401-023-02557-1 37012443 PMC10071251

[B32] Emmanouilidou E. Melachroinou K. Roumeliotis T. Garbis S. Ntzouni M. Margaritis L. (2010). Cell-produced alpha-synuclein is secreted in a calcium-dependent manner by exosomes and impacts neuronal survival. *J. Neurosci.* 30 6838–6851. 10.1523/JNEUROSCI.5699-09.2010 20484626 PMC3842464

[B33] Feiler M. Strobel B. Freischmidt A. Helferich A. Kappel J. Brewer B. (2015). TDP-43 is intercellularly transmitted across axon terminals. *J. Cell Biol.* 211 897–911. 10.1083/jcb.201504057 26598621 PMC4657165

[B34] Fevrier B. Vilette D. Archer F. Loew D. Faigle W. Vidal M. (2004). Cells release prions in association with exosomes. *Proc. Natl. Acad. Sci. U.S.A.* 101 9683–9688. 10.1073/pnas.0308413101 15210972 PMC470735

[B35] Fiandaca M. Kapogiannis D. Mapstone M. Boxer A. Eitan E. Schwartz J. (2015). Identification of preclinical Alzheimer’s disease by a profile of pathogenic proteins in neurally derived blood exosomes: A case-control study. *Alzheimers Dement.* 11:600–7.e1. 10.1016/j.jalz.2014.06.008 25130657 PMC4329112

[B36] Ganesan D. Cai Q. (2021). Understanding amphisomes. *Biochem. J.* 478 1959–1976. 34047789 10.1042/BCJ20200917 PMC8935502

[B37] García-Romero N. Carrión-Navarro J. Esteban-Rubio S. Lázaro-Ibáñez E. Peris-Celda M. Alonso M. (2017). DNA sequences within glioma-derived extracellular vesicles can cross the intact blood-brain barrier and be detected in peripheral blood of patients. *Oncotarget* 8 1416–1428. 10.18632/oncotarget.13635 27902458 PMC5352065

[B38] Gelibter S. Pisa M. Croese T. Finardi A. Mandelli A. Sangalli F. (2021). Spinal fluid myeloid microvesicles predict disease course in multiple sclerosis. *Ann. Neurol.* 90 253–265. 10.1002/ana.26154 34216397

[B39] Gillen K. Mubarak M. Park C. Ponath G. Zhang S. Dimov A. (2021). QSM is an imaging biomarker for chronic glial activation in multiple sclerosis lesions. *Ann. Clin. Transl. Neurol.* 8 877–886. 10.1002/acn3.51338 33704933 PMC8045922

[B40] Goetzl E. J. Schwartz J. B. Abner E. L. Jicha G. A. Kapogiannis D. (2018). High complement levels in astrocyte-derived exosomes of Alzheimer disease. *Ann. Neurol.* 83 544–552. 29406582 10.1002/ana.25172 PMC5867263

[B41] Goetzl E. Boxer A. Schwartz J. Abner E. Petersen R. Miller B. (2015a). Altered lysosomal proteins in neural-derived plasma exosomes in preclinical Alzheimer disease. *Neurology* 85 40–47. 10.1212/WNL.0000000000001702 26062630 PMC4501943

[B42] Goetzl E. Boxer A. Schwartz J. Abner E. Petersen R. Miller B. (2015b). Low neural exosomal levels of cellular survival factors in Alzheimer’s disease. *Ann Clin Transl Neurol.* 2 769–773. 10.1002/acn3.211 26273689 PMC4531059

[B43] Goetzl E. Kapogiannis D. Schwartz J. Lobach I. Goetzl L. Abner E. (2016a). Decreased synaptic proteins in neuronal exosomes of frontotemporal dementia and Alzheimer’s disease. *FASEB J.* 30 4141–4148. 10.1096/fj.201600816R 27601437 PMC5102122

[B44] Goetzl E. Mustapic M. Kapogiannis D. Eitan E. Lobach I. Goetzl L. (2016b). Cargo proteins of plasma astrocyte-derived exosomes in Alzheimer’s disease. *FASEB J.* 30 3853–3859. 10.1096/fj.201600756R 27511944 PMC5067254

[B45] Gomes C. Keller S. Altevogt P. Costa J. (2007). Evidence for secretion of Cu,Zn superoxide dismutase via exosomes from a cell model of amyotrophic lateral sclerosis. *Neurosci. Lett.* 428 43–46. 10.1016/j.neulet.2007.09.024 17942226

[B46] Gomes D. E. Witwer K. W. (2022). L1CAM-associated extracellular vesicles: A systematic review of nomenclature, sources, separation, and characterization. *J. Extracell. Biol.* 1:e35. 10.1002/jex2.35 PMC9045013 35492832

[B47] Gómez-Río M. Caballero M. Górriz Sáez J. Mínguez-Castellanos A. (2016). Diagnosis of neurodegenerative diseases: The clinical approach. *Curr. Alzheimer Res.* 13 469–474. 10.2174/1567205013666151116141603 26567736

[B48] Grad L. Yerbury J. Turner B. Guest W. Pokrishevsky E. O’Neill M. (2014). Intercellular propagated misfolding of wild-type Cu/Zn superoxide dismutase occurs via exosome-dependent and -independent mechanisms. *Proc. Natl. Acad. Sci. U.S.A.* 111 3620–3625. 10.1073/pnas.1312245111 24550511 PMC3948312

[B49] Grey M. Dunning C. Gaspar R. Grey C. Brundin P. Sparr E. (2015). Acceleration of α-synuclein aggregation by exosomes. *J. Biol. Chem.* 290 2969–2982. 10.1074/jbc.M114.585703 25425650 PMC4317028

[B50] Guo B. B. Bellingham S. A. Hill A. F. (2016). Stimulating the release of exosomes increases the intercellular transfer of prions. *J. Biol. Chem.* 291 5128–5137. 26769968 10.1074/jbc.M115.684258 PMC4777847

[B51] Guo M. Wang J. Zhao Y. Feng Y. Han S. Dong Q. (2020). Microglial exosomes facilitate α-synuclein transmission in Parkinson’s disease. *Brain* 143 1476–1497. 10.1093/brain/awaa090 32355963 PMC7241957

[B52] Hagey D. Ojansivu M. Bostancioglu B. Saher O. Bost J. Gustafsson M. (2023). The cellular response to extracellular vesicles is dependent on their cell source and dose. *Sci. Adv.* 9:eadh1168. 10.1126/sciadv.adh1168 37656796 PMC11629882

[B53] Hansson O. (2021). Biomarkers for neurodegenerative diseases. *Nat. Med.* 27 954–963. 10.1038/s41591-021-01382-x 34083813

[B54] Hartmann A. Muth C. Dabrowski O. Krasemann S. Glatzel M. (2017). Exosomes and the prion protein: More than one truth. *Front. Neurosci.* 11:194. 10.3389/fnins.2017.00194 28469550 PMC5395619

[B55] Helwa I. Cai J. Drewry M. Zimmerman A. Dinkins M. Khaled M. (2017). A comparative study of serum exosome isolation using differential ultracentrifugation and three commercial reagents. *PLoS One* 12:e0170628. 10.1371/journal.pone.0170628 28114422 PMC5256994

[B56] Hermann D. Peruzzotti-Jametti L. Giebel B. Pluchino S. (2024). Extracellular vesicles set the stage for brain plasticity and recovery by multimodal signalling. *Brain* 147 372–389. 10.1093/brain/awad332 37768167 PMC10834259

[B57] Hettich B. Ben-Yehuda Greenwald M. Werner S. Leroux J. (2020). Exosomes for wound healing: Purification optimization and identification of bioactive components. *Adv. Sci.* 7:2002596. 10.1002/advs.202002596 33304765 PMC7709981

[B58] Hornung S. Dutta S. Bitan G. (2020). CNS-derived blood exosomes as a promising source of biomarkers: Opportunities and challenges. *Acta Neuropathol.* 13:38. 10.3389/fnmol.2020.00038 PMC7096580 32265650

[B59] Hoshino A. Kim H. Bojmar L. Gyan K. Cioffi M. Hernandez J. (2020). Extracellular vesicle and particle biomarkers define multiple human cancers. *Cell* 182:1044–1061.e18. 10.1016/j.cell.2020.07.009 32795414 PMC7522766

[B60] Iguchi Y. Eid L. Parent M. Soucy G. Bareil C. Riku Y. (2016). Exosome secretion is a key pathway for clearance of pathological TDP-43. *Brain* 139 3187–3201. 10.1093/brain/aww237 27679482 PMC5840881

[B61] Jeppesen D. K. Zhang Q. Franklin J. L. Coffey R. J. (2023). Extracellular vesicles and nanoparticles: Emerging complexities. *Trends Cell Biol.* 33 667–681. 36737375 10.1016/j.tcb.2023.01.002 PMC10363204

[B62] Jeppesen D. Fenix A. Franklin J. Higginbotham J. Zhang Q. Zimmerman L. (2019). Reassessment of Exosome Composition. *Cell* 177:428–445.e18. 10.1016/j.cell.2019.02.029 30951670 PMC6664447

[B63] Jia L. Qiu Q. Zhang H. Chu L. Du Y. Zhang J. (2019). Concordance between the assessment of Aβ42, T-tau, and P-T181-tau in peripheral blood neuronal-derived exosomes and cerebrospinal fluid. *Alzheimers Dement.* 15 1071–1080. 10.1016/j.jalz.2019.05.002 31422798

[B64] Jiang C. Hopfner F. Katsikoudi A. Hein R. Catli C. Evetts S. (2020). Serum neuronal exosomes predict and differentiate Parkinson’s disease from atypical parkinsonism. *J. Neurol. Neurosurg. Psychiatry* 91 720–729. 10.1136/jnnp-2019-322588 32273329 PMC7361010

[B65] Joshi P. Benussi L. Furlan R. Ghidoni R. Verderio C. (2015). Extracellular vesicles in Alzheimer’s disease: Friends or foes? Focus on aβ-vesicle interaction. *Int. J. Mol. Sci.* 16 4800–4813. 10.3390/ijms16034800 25741766 PMC4394450

[B66] Joshi P. Turola E. Ruiz A. Bergami A. Libera D. Benussi L. (2014). Microglia convert aggregated amyloid-β into neurotoxic forms through the shedding of microvesicles. *Cell Death Differ.* 21 582–593. 10.1038/cdd.2013.180 24336048 PMC3950321

[B67] Jurga A. M. Paleczna M. Kuter K. Z. (2020). Overview of general and discriminating markers of differential microglia phenotypes. *Front. Cell. Neurosci.* 14:198. 10.3389/fncel.2020.00198 32848611 PMC7424058

[B68] Jy W. Minagar A. Jimenez J. Sheremata W. Mauro L. Horstman L. (2004). Endothelial microparticles (EMP) bind and activate monocytes: Elevated EMP-monocyte conjugates in multiple sclerosis. *Front. Biosci.* 9:3137–3144. 10.2741/1466 15353343

[B69] Kappos L. Bar-Or A. Cree B. Fox R. Giovannoni G. Gold R. (2018). Siponimod versus placebo in secondary progressive multiple sclerosis (EXPAND): A double-blind, randomised, phase 3 study. *Lancet* 391 1263–1273. 10.1016/S0140-6736(18)30475-6 29576505

[B70] Katsu M. Hama Y. Utsumi J. Takashina K. Yasumatsu H. Mori F. (2019). MicroRNA expression profiles of neuron-derived extracellular vesicles in plasma from patients with amyotrophic lateral sclerosis. *Neurosci. Lett.* 708:134176. 10.1016/j.neulet.2019.03.048 31173847

[B71] Kaunzner U. Kang Y. Zhang S. Morris E. Yao Y. Pandya S. (2019). Quantitative susceptibility mapping identifies inflammation in a subset of chronic multiple sclerosis lesions. *Brain* 142 133–145. 10.1093/brain/awy296 30561514 PMC6308309

[B72] Kim J. Lee H. Park K. Shin S. (2020). Rapid and efficient isolation of exosomes by clustering and scattering. *J. Clin. Med.* 9:650. 10.3390/jcm9030650 PMC7141250 32121214

[B73] Ko J. Hemphill M. Yang Z. Beard K. Sewell E. Shallcross J. (2020). Multi-dimensional mapping of brain-derived extracellular vesicle microrna biomarker for traumatic brain injury diagnostics. *J. Neurotrauma* 37 2424–2434. 10.1089/neu.2018.6220 30950328 PMC7698852

[B74] Ko J. Hemphill M. Yang Z. Sewell E. Na Y. Sandsmark D. (2018). Diagnosis of traumatic brain injury using miRNA signatures in nanomagnetically isolated brain-derived extracellular vesicles. *Lab Chip* 18 3617–3630. 10.1039/c8lc00672e 30357245 PMC6334845

[B75] Ko J. Wang Y. Sheng K. Weitz D. Weissleder R. (2021). Sequencing-based protein analysis of single extracellular vesicles. *ACS Nano* 15 5631–5638. 10.1021/acsnano.1c00782 33687214 PMC8742254

[B76] Kuhn S. Gritti L. Crooks D. Dombrowski Y. (2019). Oligodendrocytes in development, myelin generation and beyond. *Cells* 8:1424. 10.3390/cells8111424 31726662 PMC6912544

[B77] Kumar A. Su Y. Sharma M. Singh S. Kim S. Peavey J. (2023). MicroRNA expression in extracellular vesicles as a novel blood-based biomarker for Alzheimer’s disease. *Alzheimers Dement.* 19 4952–4966. 10.1002/alz.13055 37071449 PMC11663460

[B78] Lai J. Chau Z. Chen S. Hill J. Korpany K. Liang N. (2022). Exosome processing and characterization approaches for research and technology development. *Adv. Sci.* 9:e2103222. 10.1002/advs.202103222 35332686 PMC9130923

[B79] LeBleu V. S. Kalluri R. (2020). Exosomes as a multicomponent biomarker platform in cancer. *Trends Cancer* 6 767–774. 32307267 10.1016/j.trecan.2020.03.007

[B80] Lee H. J. Patel S. Lee S. J. (2005). Intravesicular localization and exocytosis of alpha-synuclein and its aggregates. *J. Neurosci.* 25 6016–6024. 15976091 10.1523/JNEUROSCI.0692-05.2005 PMC6724798

[B81] Lee H. J. Suk J. E. Bae E. J. Lee S. J. (2008). Clearance and deposition of extracellular alpha-synuclein aggregates in microglia. *Biochem. Biophys. Res. Commun.* 372 423–428. 18492487 10.1016/j.bbrc.2008.05.045

[B82] Li Y. Xia M. Meng S. Wu D. Ling S. Chen X. (2022b). MicroRNA-29c-3p in dual-labeled exosome is a potential diagnostic marker of subjective cognitive decline. *Neurobiol. Dis.* 171:105800. 10.1016/j.nbd.2022.105800 35752392

[B83] Li Y. Meng S. Di W. Xia M. Dong L. Zhao Y. (2022a). Amyloid-β protein and MicroRNA-384 in NCAM-Labeled exosomes from peripheral blood are potential diagnostic markers for Alzheimer’s disease. *CNS Neurosci. Ther.* 28 1093–1107. 10.1111/cns.13846 35470961 PMC9160455

[B84] Lim J. Choi M. Lee H. Kim Y. Han J. Lee E. (2019). Direct isolation and characterization of circulating exosomes from biological samples using magnetic nanowires. *J. Nanobiotechnol.* 17:1. 10.1186/s12951-018-0433-3 30612562 PMC6322342

[B85] Liu W. Bai X. Zhang A. Huang J. Xu S. Zhang J. (2019). Role of exosomes in central nervous system diseases. *Front. Mol. Neurosci.* 12:240. 10.3389/fnmol.2019.00240 31636538 PMC6787718

[B86] Liu W. Lin H. Lin M. Yu Y. Liu H. Dai Y. (2022). Emerging blood exosome-based biomarkers for preclinical and clinical Alzheimer’s disease: A meta-analysis and systematic review. *Neural Regen. Res.* 17 2381–2390. 10.4103/1673-5374.335832 35535875 PMC9120706

[B87] López-Pérez Ó Sanz-Rubio D. Hernaiz A. Betancor M. Otero A. Castilla J. (2021). Cerebrospinal fluid and plasma small extracellular vesicles and miRNAs as biomarkers for Prion diseases. *Int. J. Mol. Sci.* 22:6822. 10.3390/ijms22136822 34201940 PMC8268953

[B88] Lucien F. Gustafson D. Lenassi M. Li B. Teske J. Boilard E. (2023). MIBlood-EV: Minimal information to enhance the quality and reproducibility of blood extracellular vesicle research. *J. Extracell. Vesicles* 12:e12385. 10.1002/jev2.12385 38063210 PMC10704543

[B89] Mallik S. Samson R. S. Wheeler-Kingshott C. A. Miller D. H. (2014). Imaging outcomes for trials of remyelination in multiple sclerosis. *J. Neurol. Neurosurg. Psychiatry* 85 1396–1404. 24769473 10.1136/jnnp-2014-307650 PMC4335693

[B90] Malm T. Loppi S. Kanninen K. (2016). Exosomes in Alzheimer’s disease. *Neurochem. Int.* 97 193–199. 10.3390/ijms231810722 27131734

[B91] Masuda T. Sankowski R. Staszewski O. Böttcher C. Amann L. Sagar (2019). Spatial and temporal heterogeneity of mouse and human microglia at single-cell resolution. *Nature* 566 388–392. 10.1038/s41586-019-0924-x 30760929

[B92] Mathieu M. Martin-Jaular L. Lavieu G. Théry C. (2019). Specificities of secretion and uptake of exosomes and other extracellular vesicles for cell-to-cell communication. *Nat. Cell Biol.* 21 9–17. 10.1038/s41556-018-0250-9 30602770

[B93] Mavroeidi P. Vetsi M. Dionysopoulou D. Xilouri M. (2022). Exosomes in alpha-synucleinopathies: Propagators of pathology or potential candidates for nanotherapeutics? *Biomolecules* 12:957. 10.3390/biom12070957 35883513 PMC9313025

[B94] Mazzucco M. Mannheim W. Shetty S. Linden J. R. (2022). CNS endothelial derived extracellular vesicles are biomarkers of active disease in multiple sclerosis. *Fluids Barriers CNS* 19:13. 10.1186/s12987-021-00299-4 35135557 PMC8822708

[B95] Medalla M. Chang W. Calderazzo S. Go V. Tsolias A. Goodliffe J. (2020). Treatment with mesenchymal-derived extracellular vesicles reduces injury-related pathology in pyramidal neurons of monkey perilesional ventral premotor cortex. *J. Neurosci.* 40 3385–3407. 10.1523/JNEUROSCI.2226-19.2020 32241837 PMC7178914

[B96] Melo S. Luecke L. Kahlert C. Fernandez A. Gammon S. Kaye J. (2015). Glypican-1 identifies cancer exosomes and detects early pancreatic cancer. *Nature* 523 177–182. 10.1038/nature14581 26106858 PMC4825698

[B97] Meloni M. Agliardi C. Guerini F. Zanzottera M. Bolognesi E. Picciolini S. (2023). Oligomeric α-synuclein and tau aggregates in NDEVs differentiate Parkinson’s disease from atypical Parkinsonisms. *Neurobiol. Dis.* 176:105947. 10.1016/j.nbd.2022.105947 36481435

[B98] Nabhan J. Hu R. Oh R. Cohen S. Lu Q. (2012). Formation and release of arrestin domain-containing protein 1-mediated microvesicles (ARMMs) at plasma membrane by recruitment of TSG101 protein. *Proc. Natl. Acad. Sci. U.S.A.* 109 4146–4151. 10.1073/pnas.1200448109 22315426 PMC3306724

[B99] Navajas R. Corrales F. J. Paradela A. (2019). Serum exosome isolation by size-exclusion chromatography for the discovery and validation of preeclampsia-associated biomarkers. *Methods Mol. Biol.* 1959 39–50. 30852814 10.1007/978-1-4939-9164-8_3

[B100] Neuspiel M. Schauss A. Braschi E. Zunino R. Rippstein P. Rachubinski R. (2008). Cargo-selected transport from the mitochondria to peroxisomes is mediated by vesicular carriers. *Curr. Biol.* 18 102–108. 10.1016/j.cub.2007.12.038 18207745

[B101] Ngolab J. Trinh I. Rockenstein E. Mante M. Florio J. Trejo M. (2017). Brain-derived exosomes from dementia with Lewy bodies propagate α-synuclein pathology. *Acta Neuropathol. Commun.* 5:46. 10.1186/s40478-017-0445-5 28599681 PMC5466770

[B102] Nonaka T. Masuda-Suzukake M. Arai T. Hasegawa Y. Akatsu H. Obi T. (2013). Prion-like properties of pathological TDP-43 aggregates from diseased brains. *Cell Rep.* 4 124–134. 10.1016/j.celrep.2013.06.007 23831027

[B103] Norman M. Ter-Ovanesyan D. Trieu W. Lazarovits R. Kowal E. Lee J. (2021). L1CAM is not associated with extracellular vesicles in human cerebrospinal fluid or plasma. *Nat. Methods* 18 631–634. 10.1038/s41592-021-01174-8 34092791 PMC9075416

[B104] Nylander A. Hafler D. A. (2012). Multiple sclerosis. *J. Clin. Invest.* 122 1180–1188. 22466660 10.1172/JCI58649 PMC3314452

[B105] Ohmichi T. Mitsuhashi M. Tatebe H. Kasai T. Ali El-Agnaf O. M. Tokuda T. (2019). Quantification of brain-derived extracellular vesicles in plasma as a biomarker to diagnose Parkinson’s and related diseases. *Parkinsonism Relat. Disord.* 61 82–87. 10.1016/j.parkreldis.2018.11.021 30502924

[B106] Peruzzotti-Jametti L. Bernstock J. Willis C. Manferrari G. Rogall R. Fernandez-Vizarra E. (2021). Neural stem cells traffic functional mitochondria via extracellular vesicles. *PLoS Biol.* 19:e3001166. 10.1371/journal.pbio.3001166 33826607 PMC8055036

[B107] Ponath G. Park C. Pitt D. (2018). The Role of Astrocytes in Multiple Sclerosis. *Front. Immunol.* 9:217. 10.3389/fimmu.2018.00217 29515568 PMC5826071

[B108] Pounders J. Hill E. Hooper D. Zhang X. Biesiada J. Kuhnell D. (2022). MicroRNA expression within neuronal-derived small extracellular vesicles in frontotemporal degeneration. *Medicine* 101:e30854. 10.1097/MD.0000000000030854 36221381 PMC9542922

[B109] Prinz M. Erny D. Hagemeyer N. (2017). Ontogeny and homeostasis of CNS myeloid cells. *Nat. Immunol.* 18 385–392. 28323268 10.1038/ni.3703

[B110] Rajendran L. Honsho M. Zahn T. Keller P. Geiger K. Verkade P. (2006). Alzheimer’s disease beta-amyloid peptides are released in association with exosomes. *Proc. Natl. Acad. Sci. U.S.A.* 103 11172–11177. 10.1073/pnas.0603838103 16837572 PMC1544060

[B111] Rissanen E. Tuisku J. Vahlberg T. Sucksdorff M. Paavilainen T. Parkkola R. (2018). Microglial activation, white matter tract damage, and disability in MS. *Neurol. Neuroimmunol. Neuroinflamm.* 5:e443. 10.1212/NXI.0000000000000443 29520366 PMC5840890

[B112] Scaroni F. Visconte C. Serpente M. Golia M. Gabrielli M. Huiskamp M. (2022). miR-150-5p and let-7b-5p in blood myeloid extracellular vesicles track cognitive symptoms in patients with multiple sclerosis. *Cells* 11:1551. 10.3390/cells11091551 35563859 PMC9104242

[B113] Selmaj K. Mycko M. Furlan R. Rejdak K. (2022). Fluid phase biomarkers in multiple sclerosis. *Curr. Opin. Neurol.* 35 286–292. 10.1097/WCO.0000000000001058 35674070

[B114] Serpente M. Fenoglio C. D’Anca M. Arcaro M. Sorrentino F. Visconte C. (2020). MiRNA profiling in plasma neural-derived small extracellular vesicles from patients with Alzheimer’s disease. *Cells* 9:1443. 10.3390/cells9061443 32531989 PMC7349735

[B115] Shi M. Kovac A. Korff A. Cook T. Ginghina C. Bullock K. (2016). CNS tau efflux via exosomes is likely increased in Parkinson’s disease but not in Alzheimer’s disease. *Alzheimers Dement.* 12 1125–1131. 10.1016/j.jalz.2016.04.003 27234211 PMC5107127

[B116] Shi M. Liu C. Cook T. Bullock K. Zhao Y. Ginghina C. (2014). Plasma exosomal α-synuclein is likely CNS-derived and increased in Parkinson’s disease. *Acta Neuropathol.* 128 639–650. 10.1007/s00401-014-1314-y 24997849 PMC4201967

[B117] Song L. Shan D. Zhao M. Pink B. Minnehan K. York L. (2013). Direct detection of bacterial genomic DNA at sub-femtomolar concentrations using single molecule arrays. *Anal Chem.* 85 1932–1939. 10.1021/ac303426b 23331316

[B118] Song S. Lee J. Jeon M. Kim S. Lee C. Sim S. (2023). Precise profiling of exosomal biomarkers via programmable curved plasmonic nanoarchitecture-based biosensor for clinical diagnosis of Alzheimer’s disease. *Biosens. Bioelectron.* 230:115269. 10.1016/j.bios.2023.115269 37001292

[B119] Song Y. Milon B. Ott S. Zhao X. Sadzewicz L. Shetty A. (2018). A comparative analysis of library prep approaches for sequencing low input translatome samples. *BMC Genom.* 19:696. 10.1186/s12864-018-5066-2 30241496 PMC6151020

[B120] Sproviero D. Gagliardi S. Zucca S. Arigoni M. Giannini M. Garofalo M. (2022). Extracellular vesicles derived from plasma of patients with neurodegenerative disease have common transcriptomic profiling. *Front. Aging Neurosci.* 14:785741. 10.3389/fnagi.2022.785741 35250537 PMC8889100

[B121] Stefura W. Graham C. Lotoski L. HayGlass K. (2019). Improved methods for quantifying human chemokine and cytokine biomarker responses: Ultrasensitive elisa and MESO scale electrochemiluminescence assays. *Methods Mol. Biol.* 2020 91–114. 10.1007/978-1-4939-9591-2_7 31177494

[B122] Stranska R. Gysbrechts L. Wouters J. Vermeersch P. Bloch K. Dierickx D. (2018). Comparison of membrane affinity-based method with size-exclusion chromatography for isolation of exosome-like vesicles from human plasma. *J. Transl. Med.* 16:1. 10.1186/s12967-017-1374-6 29316942 PMC5761138

[B123] Taha H. Hornung S. Dutta S. Fenwick L. Lahgui O. Howe K. (2023). Toward a biomarker panel measured in CNS-originating extracellular vesicles for improved differential diagnosis of Parkinson’s disease and multiple system atrophy. *Transl. Neurodegener.* 12:14. 10.1186/s40035-023-00346-0 36935518 PMC10026428

[B124] Taylor S. Laperriere G. Germain H. (2017). Droplet digital PCR versus qPCR for gene expression analysis with low abundant targets: From variable nonsense to publication quality data. *Sci. Rep.* 7:2409. 10.1038/s41598-017-02217-x 28546538 PMC5445070

[B125] Teixeira M. Sheta R. Idi W. Oueslati A. (2021). Alpha-synuclein and the endolysosomal system in Parkinson’s disease: Guilty by association. *Biomolecule* 11:1333. 10.3390/biom11091333 34572546 PMC8472725

[B126] Thebault S. Bose G. Booth R. Freedman M. (2022). Serum neurofilament light in MS: The first true blood-based biomarker? *Mult. Scler.* 28 1491–1497. 10.1177/1352458521993066 33565908 PMC9315170

[B127] Thompson A. Gray E. Heman-Ackah S. Mäger I. Talbot K. Andaloussi S. (2016). Extracellular vesicles in neurodegenerative disease - pathogenesis to biomarkers. *Nat. Rev. Neurol.* 12 346–357. 10.1038/nrneurol.2016.68 27174238

[B128] Tomlinson P. Zheng Y. Fischer R. Heidasch R. Gardiner C. Evetts S. (2015). Identification of distinct circulating exosomes in Parkinson’s disease. *Ann. Clin. Transl. Neurol.* 2 353–361. 10.1002/acn3.175 25909081 PMC4402081

[B129] Trapp B. Bernier L. Andrews S. Colman D. (1988). Cellular and subcellular distribution of 2’,3’-cyclic nucleotide 3’-phosphodiesterase and its mRNA in the rat central nervous system. *J. Neurochem.* 51 859–868. 10.1111/j.1471-4159.1988.tb01822.x 2842456

[B130] Uhlen M. Oksvold P. Fagerberg L. Lundberg E. Jonasson K. Forsberg M. (2010). Towards a knowledge-based human protein atlas. *Nat. Biotechnol.* 28 1248–1250. 10.1038/nbt1210-1248 21139605

[B131] Veerman R. Teeuwen L. Czarnewski P. Güclüler Akpinar G. Sandberg A. Cao X. (2021). Molecular evaluation of five different isolation methods for extracellular vesicles reveals different clinical applicability and subcellular origin. *J. Extracell. Vesicles* 10:e12128. 10.1002/jev2.12128 34322205 PMC8298890

[B132] Verderio C. Muzio L. Turola E. Bergami A. Novellino L. Ruffini F. (2012). Myeloid microvesicles are a marker and therapeutic target for neuroinflammation. *Ann. Neurol.* 72 610–624. 10.1002/ana.23627 23109155

[B133] Vilette D. Laulagnier K. Huor A. Alais S. Simoes S. Maryse R. (2015). Efficient inhibition of infectious prions multiplication and release by targeting the exosomal pathway. *Cell Mol Life Sci.* 72 4409–4427. 10.1007/s00018-015-1945-8 26047659 PMC11113226

[B134] Vogelstein B. Kinzler K. W. (1999). Digital PCR. *Proc. Natl. Acad. Sci. U.S.A.* 96 9236–9241. 10430926 10.1073/pnas.96.16.9236 PMC17763

[B135] Wan R. Hussain A. Behfar A. Moran S. Zhao C. (2022). The therapeutic potential of exosomes in soft tissue repair and regeneration. *Int. J. Mol. Sci.* 23:3869. 10.3390/ijms23073869 35409228 PMC8998690

[B136] Wang C. Börger V. Sardari M. Murke F. Skuljec J. Pul R. (2020). Mesenchymal stromal cell-derived small extracellular vesicles induce ischemic neuroprotection by modulating leukocytes and specifically neutrophils. *Stroke* 51 1825–1834. 10.1161/STROKEAHA.119.028012 32312217

[B137] Wang X. Tian L. Lu J. Ng I. O.-L. (2022). Exosomes and cancer – diagnostic and prognostic biomarkers and therapeutic vehicle. *Oncogenesis* 11:54. 10.1038/s41389-022-00431-5 PMC9477829 36109501

[B138] Wang X. Yang H. Liu C. Liu K. (2023). A new diagnostic tool for brain disorders: Extracellular vesicles derived from neuron, astrocyte, and oligodendrocyte. *Front. Mol. Neurosci.* 16:1194210. 10.3389/fnmol.2023.1194210 37621405 PMC10445044

[B139] Weerakkody J. S. (2024). Photosensitive nanoprobes for rapid high purity isolation and size-specific enrichment of synthetic and extracellular vesicle subpopulations. *Adv. Funct. Mater.* 4:2400390. 10.1002/adfm.202400390 PMC11452007 39372670

[B140] Welsh J. Goberdhan D. O’Driscoll L. Buzas E. Blenkiron C. Bussolati B. (2024). Minimal information for studies of extracellular vesicles (MISEV2023): From basic to advanced approaches. *J. Extracell. Vesicles* 13:e12404. 10.1002/jev2.12404 38326288 PMC10850029

[B141] Wingerchuk D. M. Lucchinetti C. F. (2022). Neuromyelitis optica spectrum disorder. *N. Engl. J. Med.* 387 631–639. 36070711 10.1056/NEJMra1904655

[B142] Winston C. Goetzl E. Akers J. Carter B. Rockenstein E. Galasko D. (2016). Prediction of conversion from mild cognitive impairment to dementia with neuronally derived blood exosome protein profile. *Alzheimers Dement.* 3 63–72. 10.1016/j.dadm.2016.04.001 27408937 PMC4925777

[B143] Xie Y. Chen B. Wang Q. Chen X. Lai W. Xu Y. (2023). Astrocyte-derived exosomes contribute to pathologies of neuromyelitis optica spectrum disorder in rodent model. *Ann. Neurol.* 94 163–181. 10.1002/ana.26650 36966488

[B144] Xu X. Iqbal Z. Xu L. Wen C. Duan L. Xia J. (2024). Brain-derived extracellular vesicles: Potential diagnostic biomarkers for central nervous system diseases. *Psychiatry Clin. Neurosci.* 78 83–96. 10.1111/pcn.13610 37877617

[B145] Yang D. Zhang W. Zhang H. Zhang F. Chen L. Ma L. (2020). Progress, opportunity, and perspective on exosome isolation – efforts for efficient exosome-based theranostics. *Theranostics* 10 3684–3707. 10.7150/thno.41580 32206116 PMC7069071

[B146] Yang Z. Atiyas Y. Shen H. Siedlik M. Wu J. Beard K. (2022). Ultrasensitive single extracellular vesicle detection using high throughput droplet digital enzyme-linked immunosorbent assay. *Nano Lett.* 22 4315–4324. 10.1021/acs.nanolett.2c00274 35588529 PMC9593357

[B147] Younas N. Fernandez Flores L. Hopfner F. Höglinger G. Zerr I. (2022). A new paradigm for diagnosis of neurodegenerative diseases: Peripheral exosomes of brain origin. *Transl. Neurodegener.* 11:28. 10.1186/s40035-022-00301-5 35527262 PMC9082915

[B148] Yu Z. Shi M. Stewart T. Fernagut P. Huang Y. Tian C. (2020). Reduced oligodendrocyte exosome secretion in multiple system atrophy involves SNARE dysfunction. *Brain* 143 1780–1797. 10.1093/brain/awaa110 32428221 PMC7296853

[B149] Yuyama K. Sun H. Mitsutake S. Igarashi Y. (2012). Sphingolipid-modulated exosome secretion promotes clearance of amyloid-β by microglia. *J. Biol. Chem.* 287 10977–10989. 10.1074/jbc.M111.324616 22303002 PMC3322859

[B150] Yuyama K. Sun H. Sakai S. Mitsutake S. Okada M. Tahara H. (2014). Decreased amyloid-β pathologies by intracerebral loading of glycosphingolipid-enriched exosomes in Alzheimer model mice. *J. Biol. Chem.* 289 24488–24498. 10.1074/jbc.M114.577213 25037226 PMC4148874

[B151] Zetterberg H. Schott J. M. (2019). Biomarkers for Alzheimer’s disease beyond amyloid and tau. *Nat. Med.* 25 201–203. 30728536 10.1038/s41591-019-0348-z

[B152] Zhang H. Freitas D. Kim H. Fabijanic K. Li Z. Chen H. (2018). Identification of distinct nanoparticles and subsets of extracellular vesicles by asymmetric flow field-flow fractionation. *Nat. Cell Biol.* 20 332–343. 10.1038/s41556-018-0040-4 29459780 PMC5931706

[B153] Zhang X. Yao L. Meng Y. Li B. Yang Y. Gao F. (2023). Migrasome: A new functional extracellular vesicle. *Cell Death Discov.* 9:381. 10.1038/s41420-023-01673-x 37852963 PMC10584828

[B154] Zhang Q. Jeppesen D. K. Higginbotham J. N. Franklin J. L. Coffey R. J. (2023). Comprehensive isolation of extracellular vesicles and nanoparticles. *Nat. Protoc.* 18 1462–1487. 36914899 10.1038/s41596-023-00811-0 PMC10445291

[B155] Zhao H. Yang L. Baddour J. Achreja A. Bernard V. Moss T. (2016). Tumor microenvironment derived exosomes pleiotropically modulate cancer cell metabolism. *Elife* 5:e10250. 10.7554/eLife.10250 26920219 PMC4841778

[B156] Zhao Z. Chen Z. Zhou R. Zhang X. Ye Q. Wang Y. (2019). Increased DJ-1 and α-synuclein in plasma neural-derived exosomes as potential markers for Parkinson’s disease. *Front. Aging Neurosci.* 10:438. 10.3389/fnagi.2018.00438 30692923 PMC6339871

[B157] Zhong J. Ren X. Liu W. Wang S. Lv Y. Nie L. (2021). Discovery of novel markers for identifying cognitive decline using neuron-derived exosomes. *Front. Aging Neurosci.* 13:696944. 10.3389/fnagi.2021.696944 34512304 PMC8427802

[B158] Zou J. Guo Y. Wei L. Yu F. Yu B. Xu A. (2020). Long noncoding RNA POU3F3 and α-synuclein in plasma L1CAM exosomes combined with β-glucocerebrosidase activity: Potential predictors of Parkinson’s disease. *Neurotherapeutics* 17 1104–1119. 10.1007/s13311-020-00842-5 32236821 PMC7609611

